# A novel strategy of combining abdominal surgery and endoscopy for the quick hemostasis of acute duodenal ulcer bleeding: a case report

**DOI:** 10.1186/s40792-023-01794-6

**Published:** 2024-01-02

**Authors:** Bixian Luo, Han Liu, Weihua Gong

**Affiliations:** Department of Surgery, Second Affiliated Hospital of School of Medicine, Zhejiang University, Jiefang Road #88, Hangzhou, Zhejiang 310009 People’s Republic of China

**Keywords:** Endoscopy, Laparotomy, Hemostasis, Suture, Acute duodenal ulcer bleeding

## Abstract

**Background:**

Uncontrolled ulcer bleeding of duodenal ulcer (DU) after endoscopic therapy often needs surgery. At present, cutting the bottom of the ulcer with ligation and performing its excision-lesion are the common ways to achieve immediate efficacy in stopping bleeding. For the problem of its great trauma, we seek an easy and useful technical method to reach the same therapeutic effect to stop acute bleeding.

**Methods:**

We determined the distribution of the lesion and its innervated blood vessels under the guidance of the endoscopy and then performed suture and hemostasis on the external surface of the stomach and duodenum.

**Results:**

An immediate efficacy in stopping bleeding was shown and the hemoglobin (Hb) level returned to normal after operation with no recurrence of bleeding.

**Conclusion:**

We created a successful and novel strategy for laparotomy-endoscopic assisted suture for DU emergency hemostasis without duodenectomy.

## Introduction

Gastroduodenal ulcer bleeding is one of the main causes of gastrointestinal bleeding, accounting for about 50% [1, 2]. DUs are more common than gastric ulcers (GUs) and 75% of ulcer bleeding happens in the duodenal bulb [3, 4]. The bleeding site of GU is mostly located on the minor curved side, while DU bleeding is largely located in the posterior wall of the bulb [5]. The ulcer erodes the gastroduodenal artery or the superior pancreaticoduodenal artery and its branches, resulting in bleeding [6]. Generally, uncontrolled ulcer bleeding after endoscopic therapy is treated surgically. So far, surgical intervention usually adopts two methods. One is to cut the bottom of the ulcer and carry out the operation through a suture of the ulcer to stop the bleeding; for the other, distal subtotal gastrectomy of both the bleeding lesion and ulcer lesion excision is performed to achieve a radical medical cure [7]. However, for patients who cannot tolerate gastrectomy of surgical strike and seek quick hemostasis, simple ligation of gastroduodenal vessels outside the body to stop bleeding is a good strategy.

## Case report

### Endoscopic hemostasis: attempt and failure

**Fig. 1:**
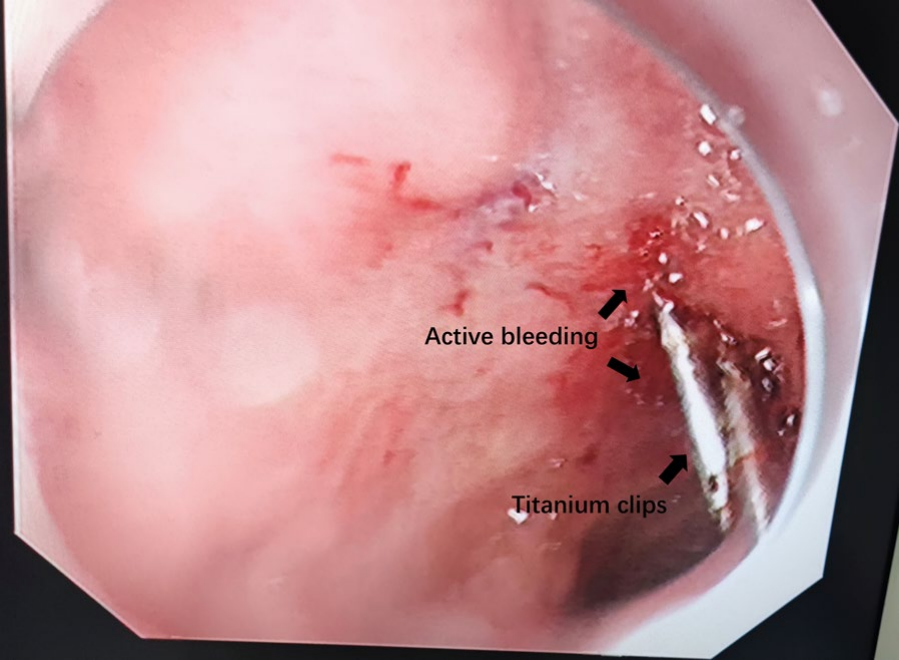
The titanium clips we used and the location of active bleeding area

We conducted an exploratory and prospective surgical strategy of a 52-year-old male patient with active duodenal ulcer bleeding. The patient had a medical history of type 2 diabetes and partial left lobectomy was performed because of lung infections. The results of vital signs were at normal levels after performing a thorough physical examination. We firstly measured the amount of Hb in the blood, which had a detection value of 123 g/L (> 90 g/L). After the effect of conservative treatment was not obvious, we immediately carried out endoscopic hemostasis. Endoscopic examination found no obvious bleeding in the gastric mucosa. When it came to the duodenum, we found that there were ulcers at both the anterior and posterior walls. There was a 6 mm white mass on the posterior wall of the duodenal bulb without active bleeding. However, a white mass about 1 cm in size was visible on the anterior wall of the duodenal bulb, covered with blood clots, and active blood oozing was obvious. The rate of active bleeding was not slowed down after 1.5 mL of 1:10,000 epinephrine was injected into both the oral and anal side in minor and major curves of the anterior bulbous wall. Though titanium clips were subsequently used to stop bleeding, the patient still had active bleeding and the peripheral Hb level measured continued to decrease to 40 g/L (Fig. 1). The endoscopic diagnosis was made: multiple ulcers with bleeding in the duodenal bulb and Forrest Ib (ulcer bleeding classification), which means seeking surgical ways of hemostasis was essential. Therefore, an emergency exploratory laparotomy was performed to find potential abdominal bleeding lesions.

### Surgical intervention: laparotomy-endoscopic assisted vessel suture

For patients who seek quick hemostasis, simple ligation of gastroduodenal vessels outside the body to stop bleeding is a good and novel strategy. Therefore, we decided to make a right upper abdominal incision and isolate the mesangial tissue, exposing the gastric antrum and duodenal bulb.

**Fig. 2:**
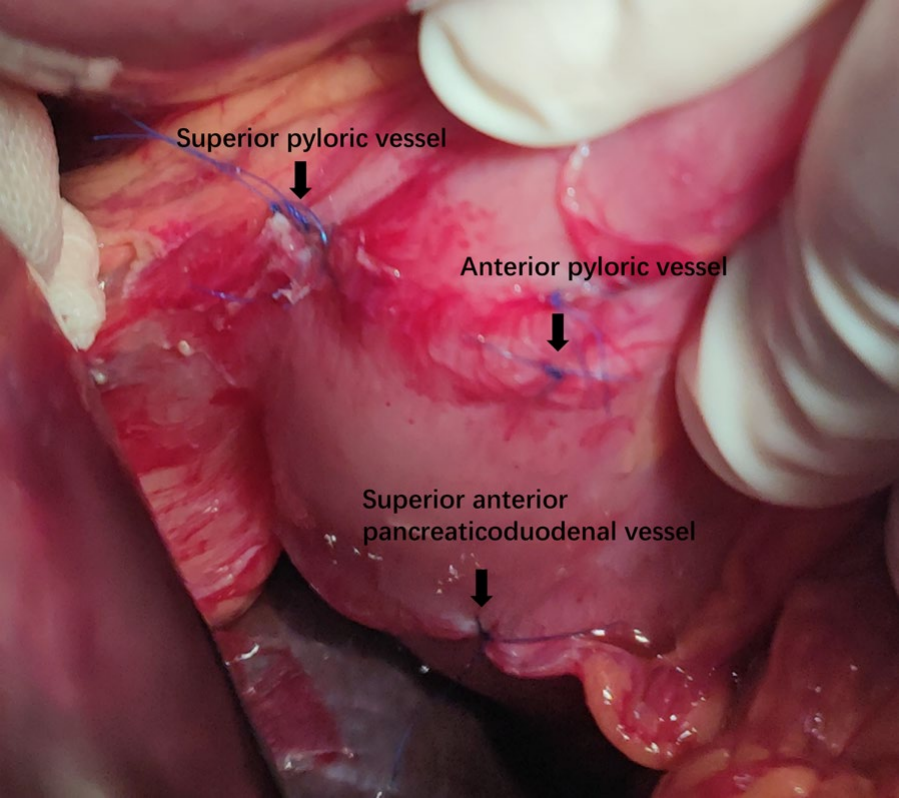
The vessels we ligated at the pyloroduodenum in the process of surgery

**Fig. 3:**
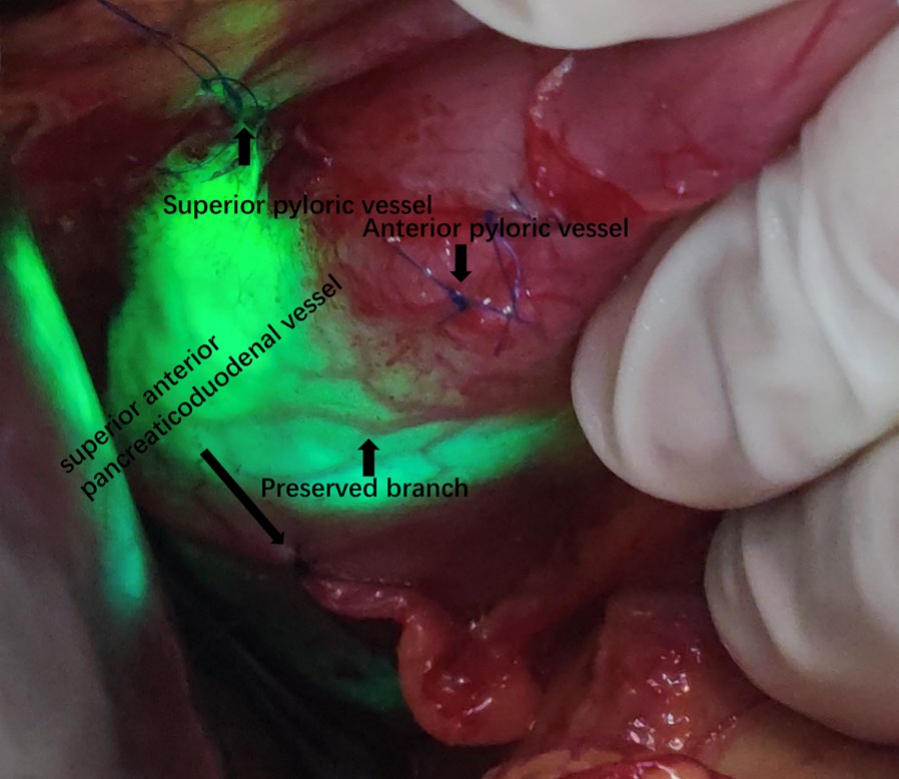
The preserved branch vessel at the duodenum under the background of endoscopic intensive light

**Fig. 4:**
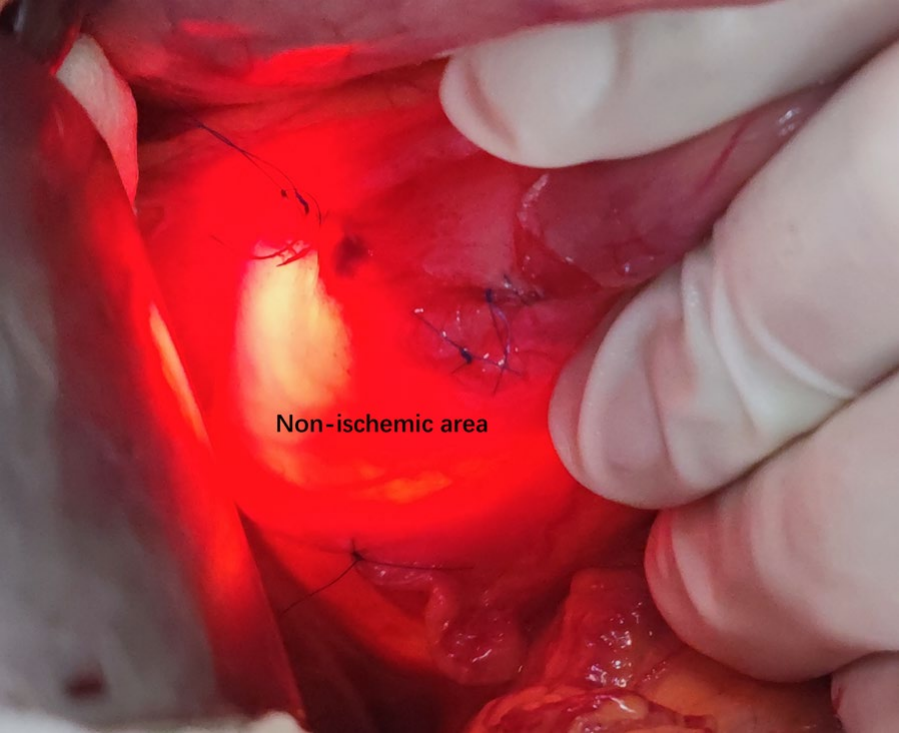
The non-ischemic area at the duodenum under the background of endoscopic intensive light

We ligated the pyloric vessels supplying the gastric antrum and duodenal bulb including the superior pyloric vessel and anterior pyloric vessel, but active bleeding still was observed under the view of endoscopy (Fig. 2). We continued to search for the culprit vessels and ligated the branch of the pancreaticoduodenal vessel artery (superior anterior pancreaticoduodenal vessel), which stopped the bleeding immediately (Fig. 2). After making sure that there was no active bleeding, we did not ligate the other vessels to avoid ischemia of the duodenum. When the shadowless lamp was turned off, the preserved blood vessels are clearly displayed under the strong light of the endoscope to find out its preserved vessels, the blood-supplied area, and the presence of the non-ischemic area (Figs. 3, 4).

### Results and benefits

As shown above, we successfully determined its culprit blood vessels (superior anterior pancreaticoduodenal vessel) and the distribution of the lesion under the guidance of the endoscope and then performed surface suture hemostasis. In the process of surgery, no blood transfusion was performed, and the amount of blood lost was estimated at 20 mL. After the operation, the Hb content in the blood gradually returned to a normal level, the postoperative occult blood test was negative for three consecutive days, and there were no postoperative complications, which all means that our technical hemostasis was successful. The benefits of our surgical techniques are shown as follows: the exact hemostasis under the direct vision of the endoscope avoided the difficult suture hemostasis of the traditional enterotomy and gastrotomy of ulcer, greatly reduced the difficulty and time needed for the operation, and avoided the possibility of various surgical complications. In addition, the necessary blood vessels can be preserved to prevent the occurrence of duodenum ischemia.

## Discussion

Endoscopic therapy is recommended for non-venous upper gastrointestinal bleeding in patients with upper gastrointestinal bleeding of the modified Forrest grading Ia–IIb [8, 9]. The patient in our study was shown to still have active bleeding after endoscopic therapy, which requires surgical intervention. Vascular embolization can be another common way used for hemostasis in addition to both endoscopic hemostasis and surgical treatment. However, the implementation of this method requires angiography to determine the bleeding vessels and then the absorbable gelatin sponge is used to stop bleeding, which needs relatively large vessels and takes a relatively long time. When the level of peripheral blood globin in patients is only 40 g/L, emergency surgical hemostasis is obviously the first choice to save patient’s life. It is worth mentioning that ICG (indocyanine green) angiography may be one good way used for identifying the bleeding vessels. However, the observation areas of blood vessels must be within the scope of microscope vision, and therefore, the surgical field should be fully exposed without any obstruction. Under this circumstance, we could try this novel strategy.

In our study, we should pay more attention to some important details to make the surgery successful, which can accumulate clinical experience and prepare for similar situations happening in the future. On one hand, Excessive loss of blood and the failure of endoscopic hemostasis caused the very low Hb and albumin levels in this situation and the increased clean ascites could be seen after entering the abdominal cavity. After repeated endoscopy hemostasis and air inflation, it was easy to lead to thinning of the intestinal wall, coupled with gas swelling and edema because of air and fluid accumulation in the gastrointestinal tract and hypoalbuminemia. Therefore, surgical incision of the skin to expose the abdominal cavity should be very careful to prevent hurting the intestinal wall; On the other hand, after opening the abdominal cavity during the operation, we need to turn off the shadowless lamp to make the related vessels and the bleeding lesions could be seen clearly under the intense light stimulated by the endoscope. The visual field could have a stronger ratio and clearly show the lesions because of the relative darkness of the abdominal cavity.

We aimed to report this study in order to introduce the following advantages of using the laparotomic-endoscopy assisted vessel suture of the duodenum. On one hand, the patient in our study had an ulcer in the anterior wall of the duodenum with ineffective endoscopic hemostasis and continued active bleeding even using titanium clips to stop bleeding (Fig. 1). It was difficult to find and suture effectively the active bleeding vessels by conventional incision and all layer-suture. Using our way of external suture dealing with the culprit vessels not only reduced the time used and its difficulty in operation but also made the patient recover faster without great trauma. On the other hand, the gastroduodenal body surface vessels enter the inner wall from the proximal serous outer layer to the inner mucosal layer nourishing each layer of gastroduodenal tissue. The submucosal vessels in the inner wall of the gastroduodenal body wall are terminal vessels. The hemostatic effect achieved by using several titanium clips to clamp the inner wall submucosal vessels under conventional endoscopic hemostasis is not as good as that achieved by the proximal vascular clamp. At the same time, the vessels on the surface of the duodenum can be clearly visible under the intensive light of the endoscope, and the ligation location can be selected according to the degree of bleeding of the culprit vessels. For small amounts of bleeding, the small terminal vessels at the distal end can be ligated firstly, while for large amounts of bleeding, the thick stem of the proximal end can be ligated to minimize postoperative gastroduodenal wall ischemic area. As discussed above, we concluded that laparoscopic endoscopic assisted sutures were the quick, effective, selective, and less invasive surgical techniques to stop bleeding. Therefore, we thought that indications for this strategy may be the patients who cannot tolerate the great trauma of gastroduodenectomy after the endoscopic hemostasis failed. However, the disadvantages of the laparotomy-endoscopic method were obvious. On one hand, if too many vessels were ligated, the duodenal wall might happen late ischemia. If too few, the bleeding could not be stopped. On the other hand, this surgical strategy had the risks of unclear exposure of the surgical field, false and failure of ligation, ligation dislodgement and so on.

About one month later, we conducted the postoperative hemoglobin and endoscopic check again, and found that the hemoglobin was at the normal level and the endoscope showed no bleeding signs in the duodenal ulcer, indicating that the emergency operation was successful and there was no recurrence. However, as for the recurrence rate of the surgical method of emergency hemostasis adopted in this paper, we intend to continue the study and will get the research data and conclusions in the future.

## Data Availability

The original contributions presented in the study are included in the article. Further inquiries can be directed to the corresponding author.

## Abbreviations

DU: Duodenal ulcer
GU: Gastric ulcer
Hb: Hemoglobin
